# Investigation of Biocrude Production from Spirulina via Catalytic Hydrothermal Liquefaction with the Ru/ZrO_2_–SiO_2_ Catalyst

**DOI:** 10.1021/acsomega.4c07632

**Published:** 2025-01-06

**Authors:** Shuai Lu, Shaobo Wang, Jinglai Zhang, Guang Xian, Yichi Qian, Hongbiao Du

**Affiliations:** † School of Ecology and Environment, Renmin University of China, No.59 Zhongguancun Street, Beijing 100872, China; ‡ Army Logistics Academy, Chongqing 401331, China; § School of Chemistry, Cardiff University, Cardiff CF10 3AT, U.K.; ∥ School of Environment, Tsinghua University, Beijing 100084, China

## Abstract

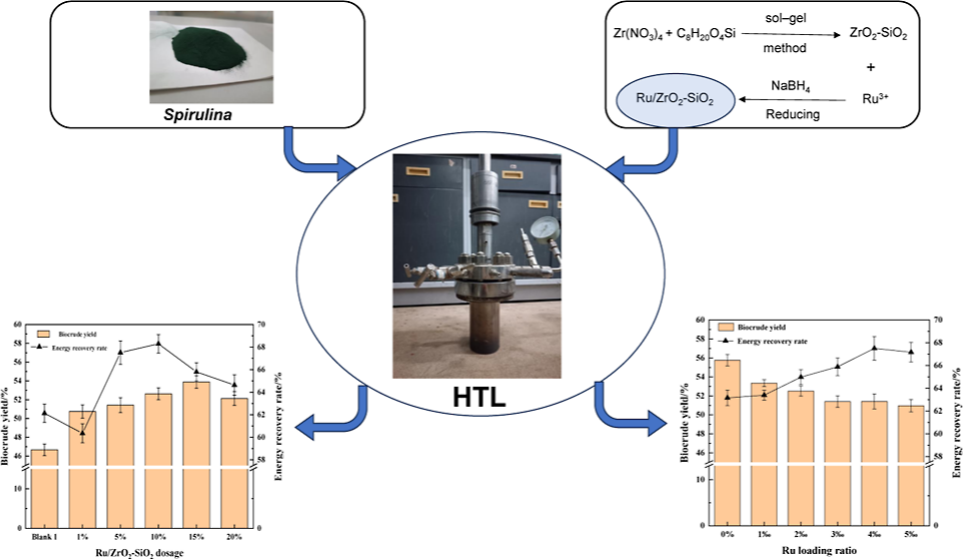

Hydrothermal liquefaction (HTL) is a promising technology for converting wet biomass to liquid fuels. However, the biocrude yield and quality in this process are unsatisfactory without catalysts. Herein, a Ru/ZrO_2_–SiO_2_ catalyst was prepared with the NaBH_4_ reducing method for the HTL of *Spirulina*. The results demonstrated the successful deposition of Ru particles onto the ZrO_2_–SiO_2_ carrier, resulting in improved crystallinity and a more stable internal structure. The effect of different loading ratios of Ru particles on biocrude production was investigated. The findings revealed a gradual decrease in biocrude yield with increasing loading ratio of Ru particles. Meanwhile, the energy recovery (ER) rate exhibited a trend of first increasing and then decreasing, with a peak of 67.51% observed at a 4‰ loading molar ratio. The GC–MS results indicated an increase in hydrocarbon content, accompanied by a decrease in carboxylic acids and esters, demonstrating the hydrodeoxygenation effect of Ru. Additionally, the impact of the Ru/ZrO_2_–SiO_2_ dosage on the HTL of *Spirulina* was examined. The highest biocrude yield of 53.89% was attained with a 15% catalyst dosage, while the highest ER rate of 68.29% was observed at a 10% catalyst dosage.

## 1 Introduction

The global demand for energy is growing rapidly with the development of social economy and industrialization, making energy a crucial factor for countries worldwide.^1^ Consequently, the limited reserves and environmental pollution associated with fossil fuels have prompted researchers to explore new energy resources. Biomass, derived from organisms formed through photosynthesis, such as trees, crops, and algae, has gained significant attention owing to its environmental friendliness, ubiquity, renewability, and neutral carbon emissions.^2^ Among the various biomass types, microalgae, considered a third-generation biomass, offer advantages, such as fast growth, high yield, and CO_2_ fixation capabilities. Moreover, microalgae can be cultivated in various water sources, including freshwater, saltwater, even wastewater, without competing with food crops for cultivated land, making them an ideal choice for energy production.^3^

The conversion of biomass into bioenergy can be achieved through various methods, including pyrolysis and hydrothermal liquefaction (HTL). Pyrolysis involves the direct thermal decomposition of biomass at high temperatures (400–1000 °C) to produce biocrude.^4^ This process, however, requires energy-intensive operations, high reaction temperatures, and a biomass dehydration procedure prior to pyrolysis. On the other hand, HTL technology utilizes sub- or supercritical water at temperatures of 250–400 °C and pressures of 5–35 MPa to convert biomass into liquid biocrude.^5,6^ Compared to pyrolysis, HTL offers energy efficiency due to its moderate reaction conditions and the ability to utilize wet biomass directly without dehydration pretreatment.^7^ These features allow for higher ER and make HTL a promising technology in the field of new energy.

Despite the advantages of HTL, the suboptimal yield and quality of the produced biocrude remain notable challenges. To address this issue, researchers did a lot of work and found that the HTL process can be influenced by many factors, such as feedstock, temperature, retention time, pressure, solid–liquid ratio, solvent, catalyst, and so on. Relevant studies were summarized in Table 1. Process optimization, seeking suitable feedstocks and reaction conditions, was the dominant method in the early stage of the HTL field. Now, more attention is being paid to catalyst preparation and application with the aim of improving the biocrude yield and quality. Catalysts used in the HTL can be classified into two categories: homogeneous and heterogeneous. Homogeneous catalysts, including acids and alkalis, have demonstrated their efficacy in enhancing biocrude oil production. For instance, Koley et al. reported that CH_3_COOH achieved the highest biocrude yield of 45% among acidic catalysts,^16^ while Zhang et al. found positive effects on biocrude yield with KOH and acetic acid catalysts.^17^ In contrast, heterogeneous catalysts offer advantages, such as easy separation, recycling, and resistance for harsh reaction conditions. Commonly used heterogeneous catalysts include activated carbon and its supported counterparts, various metal oxides and their derivatives, and molecular sieves, along with their loaded forms. Duan and Savage. investigated the effect of metal catalysts supported on carbon, such as Pd/C, Pt/C, and Ru/C, on the HTL of *Nannochloropsis* sp, resulting in significant improvements in biocrude yield from 35% to 45%.^18^ Liu et al. explored various metallic oxides, like ZrO_2_, Al_2_O_3_, TiO_2_, ZnO, MgO, and CaO, as catalysts and noted a slight reduction in biocrude yield compared to the control group. This reduction was attributed to the promotion of fatty acid formation, amide dehydration, and a weakening of the Maillard reaction between reducing sugars and amino compounds, resulting in a lower conversion rate of protein and carbohydrate to biocrude.^19^ Furthermore, Xu et al. studied the effect of Ce/HZSM-5 on the HTL of *Chlorella pyrenoidosa*, resulting in a significant increase in biocrude yield and improved quality, as evidenced by higher C and H content and a decrease in N content.^20^ Collectively, these studies provide valuable insights into the potential of different catalysts in HTL processes.

**Table 1:** Influencing Factors of the HTL Process in Producing Biocrude

feedstock	reaction conditions	catalyst	biocrude yield	ref
microalgae (*Spirulina*)	310–370 °C 15 min TS:20% water batch		30–40%	(8)
human feces	300 °CTS:15% water continuous		28%	(9)
microalgae (*Chlorellavulgaris* and *Nannochloropsisgaditana*)	350 °C 15 min water batch and continuous		batch 42.5% and 47.9% continuous 36.2% and 31.5%	(10)
sewage sludge	330 °C 30 min water/aqueous phasebatch		17.9–30.5%	(11)
oil palm empty fruit bunch	300–350 °C 20–46.7 min water/ethanol continuous	formic acid	13.4–67%	(12)
wheat straw	350–400 °C 15 min TS:20% water batch	K_2_CO_3_	23.50–32.34%	(13)
microalgae(*Spirulina*)	300 °C 30 min TS:20% water batch	ZrO_2_–SiO_2_	maximum 55.8%	(14)
chinese herb residue	320 °C 10 min TS:6.25% water batch	ZSM-5	20.8%	(15)

In our previous study,^14^ we focused on utilizing the ZrO_2_–SiO_2_ complex oxide to improve biocrude yield during HTL of *Spirulina*. Nevertheless, this process led to a deterioration in biocrude quality, characterized by an increased O content of an octahedral element and a decreasing C and H contents. To address this challenge, it is necessary to load active components onto the ZrO_2_–SiO_2_ carrier. Transition-metal elements have garnered significant attention due to their structural characteristics and catalytic properties. For instance, Liu et al. investigated HTL of *Spirulina* with various metal/C catalysts and found that Rh/C exhibited the highest bio-oil yield of 50.98% and HHV of 30.67 MJ kg^–1^.^21^ Among the transition metals, ruthenium(Ru) is an ideal choice as the loading component. First, its complex electronic structure and diverse oxidation states enhance its effectiveness in various reaction environments, which is crucial considering the complexity of the HTL process. Additionally, the catalytic hydrogenation capability of Ru contributes to improved biocrude quality in HTL systems, which is important for the subsequent utilization of biocrude.^22,23^

This study focuses on the preparation and characterization of the Ru/ZrO_2_–SiO_2_ catalyst by depositing Ru particles on the ZrO_2_–SiO_2_ carrier with the NaBH_4_ reducing method. The research aims to investigate the optimal loading ratio of Ru particles and the dosage of the Ru/ZrO_2_–SiO_2_ catalyst for HTL of *Spirulina*, with the goal of enhancing the biocrude yield and improving ER efficiency. Additionally, the study will evaluate the impact of the Ru/ZrO_2_–SiO_2_ catalyst on the composition and properties of the resulting biocrude.

## 2 Materials and Methods

### 2.1 Materials

The *Spirulina* powder utilized in this experiment was procured from Shandong Binzhou Tianjian Biotechnology Co., Ltd., and its characteristics are listed in Table 2. Tetraethyl orthosilicate (C_8_H_12_O_8_Si), ruthenium trichloride (RuCl_3_), glacial acetic acid (CH_3_COOH), hydrochloric acid (HCl), ammonia (NH_3_·H_2_O), sodium borohydride (NaBH_4_), and dichloromethane (CH_2_Cl_2_, DCM) were purchased from Sinopharm Chemical Reagent Co., while Zr(NO_3_)_4_·5H_2_O was obtained from Shanghai Macklin Biochemical Co., Ltd. All of the chemical reagents mentioned above were of analytical purity.

**Table 2:** Composition of the Raw Material (*Spirulina*)

composition	content
organic matter (wt %)	
carbohydrate	11
protein	65
lipid	6
others	5.3
element content (%,daf^a^)	
C	49.4
H	6.9
N	11.8
S	0.7
O^b^	31.2
proximate analysis (wt %)	
moisture	6.2
ash	6.5
HHV (MJ kg^–1^)	23.2

a Daf: dry ash free.

b Calculated by differences.

### 2.2 Catalyst Preparation and Characterization

The ZrO_2_–SiO_2_ complex oxide carrier was prepared by the sol–gel method, and the detailed steps can be found in our previous study.^14^ A standard solution containing 0.1 g mL^–1^ of Ru^3+^ was prepared, and concentrated hydrochloric acid was added to prevent the hydrolysis of Ru^3+^. A certain amount of the ZrO_2_–SiO_2_ catalyst carrier was placed in deionized water and thoroughly stirred. Then, the Ru^3+^ standard solution was added to the mixture, and a suitable amount of NaBH_4_ solution was added drop by drop to reduce the number of Ru^3+^ ions. Given that NaBH_4_ is sensitive to heat and can decompose at high temperatures to produce hydrogen—which is both flammable and explosive—it is crucial to avoid open flames and high-temperature sources when preparing the solution. When NaBH_4_ is introduced into water, it should be added slowly while stirring to prevent splashing caused by vigorous reactions and to minimize bubble formation. Stirring was continued for an additional 1 h after dropping to ensure a complete reaction. The catalyst was filtered with a solvent filter, and the solid part was recovered and washed several times. Next, the solids were ground in ethanol, and the Ru/ZrO_2_–SiO_2_ catalyst was obtained after drying and sieving through a 60-mesh sieve.

The crystal structure of the catalysts was determined with a Bruker (D8 Advance) X-ray diffractometer. The conventional wide-angle test was performed under CuK_α_ radiation (λ = 1.5418 °A, voltage = 40 kV, current = 150 mA), scanning the range of 2–90° at a speed of 2° min^–1^. For the BET, SEM, and XPS tests of the catalysts, Quantachrome (Autosorb IQ), ZEISS (GeminiSEM 300), and Thermofisher (Escalab 250Xi) instruments were used, respectively. Before the BET test, the catalysts were pretreated under vacuum at 300 °C for 8 h.

### 2.3 HTL Process and Biocrude Characterization

The hydrothermal liquefaction of *Spirulina* was conducted in a 500 mL batch reactor (GSH-0.5, Weihai Chemical Machinery Co., Ltd., China) heated by an external electric furnace. Prior to the HTL process, 30 g of *Spirulina*, 120 mL of deionized water, and varying amounts of catalysts were added to the reactor under a purged nitrogen atmosphere to maintain an inert environment. The reactor was then heated to 300 °C with a heating rate of 6 °C min^–1^ and stirred at 130 rpm for 30 min. After the reaction was completed, the reactor was cooled to room temperature with a tap water circulating cooling system. The mixture was subsequently rinsed with DCM, which was chosen as the solvent due to its ability to provide high biocrude yield and ER value.^24,25^ The mixture was then separated with a solution filter, followed by further separation of the liquid phase with a separatory funnel, to obtain the DCM phase. The DCM phase was evaporated for 40 min at 40 °C under negative pressure to obtain the biocrude. Each test was conducted in triplicate to ensure repeatability.

The biocrude yield (%), HHV (MJ kg^–1^), and ER (%) were calculated according to Formulas 1, 2, and 3(26)
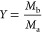
1
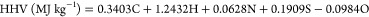
2
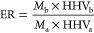
3where *Y* and *M* represent the biocrude yield and mass; the subscript “b” and “a” refer to biocrude and algae (*Spirulina*); and C, H, O, N, and S are the mass percentages of carbon, hydrogen, oxygen, nitrogen, and sulfur in the biocrude, respectively.

The elemental analysis and GC–MS characteristics can be referred to in our previous study.^14^

## 3 Results and Discussion

### 3.1 Characterization of the Ru/ZrO_2_–SiO_2_ Catalyst

#### 3.1.1 Element Information on the Ru/ZrO_2_–SiO_2_ Catalyst

**Figure 1:**
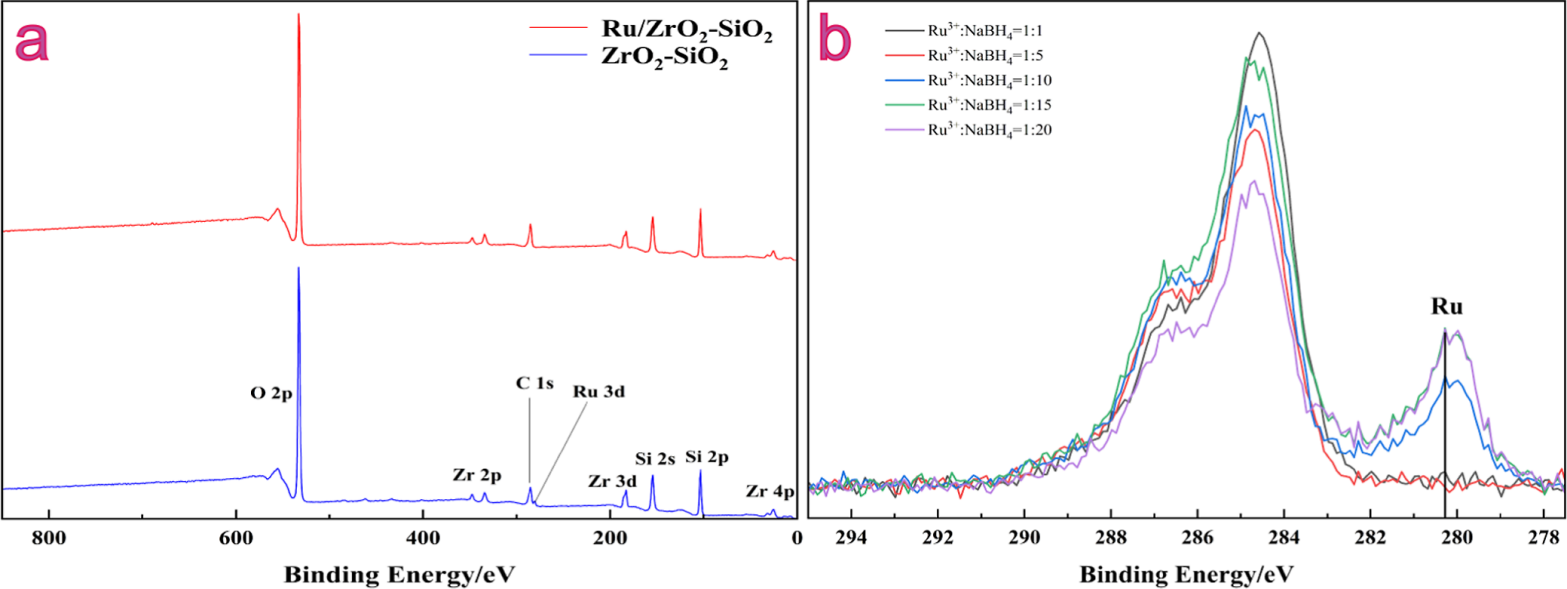
XPS (a) full spectra of Ru/ZrO_2_–SiO_2_ and ZrO_2_–SiO_2_ and (b) spectra of Ru/ZrO_2_–SiO_2_ at different reducing agent ratios.

The X-ray photoelectron spectroscopy (XPS) analysis was conducted to determine the elemental composition of the Ru/ZrO_2_–SiO_2_ catalysts. The XPS survey spectra of Ru/ZrO_2_–SiO_2_ and ZrO_2_–SiO_2_ are shown in Figure 1a. The peaks of each element in the composite oxide carriers were partially shifted compared to pure ZrO_2_ or SiO_2_, indicating internal structure changes in the coordination of ZrO_2_ and SiO_2_ caused by atomic doping.^27−29^ However, the absence of significant changes in the peaks of the elements in Ru/ZrO_2_–SiO_2_ compared to ZrO_2_–SiO_2_ demonstrated that the Ru particles did not chemically interact with the carrier but were only adsorbed and enriched on the carrier surface.^30^

The Ru loading ratio was 5‰, and the XPS range was set between 278 and 295 eV. As shown in Figure 1b, increasing the amount of NaBH_4_ led to a decrease in the ratio of Ru^3+^/NaBH_4_, resulting in a corresponding increase in Ru loading until it reached a steady state at a molar ratio of Ru^3+^/NaBH_4_ = 1:15. Further increase in NaBH_4_ did not affect the Ru loading amount, indicating that the optimal reducing agent ratio for this experiment was Ru^3+^/NaBH_4_ = 1:15. The relatively sharp and symmetrical 3d_5/2_ peak of elemental Ru suggested excellent dispersion of Ru on the ZrO_2_–SiO_2_ carrier, resulting in a relatively uniform structure.^31^ Consequently, RuCl_3_ can only be loaded after being reduced to metallic Ru(0), since the low lattice energy of RuCl_3_ made it difficult for RuCl_3_ to be enriched on the carrier surface. The binding energy of the Ru 3d5/2 peak remained unchanged after loading, indicating that no electron transfer or chemical interaction took place between the Ru metal and the ZrO_2_–SiO_2_ carrier.^32^

#### 3.1.2 Crystal Structure of the Ru/ZrO_2_–SiO_2_ Catalyst

**Figure 2:**
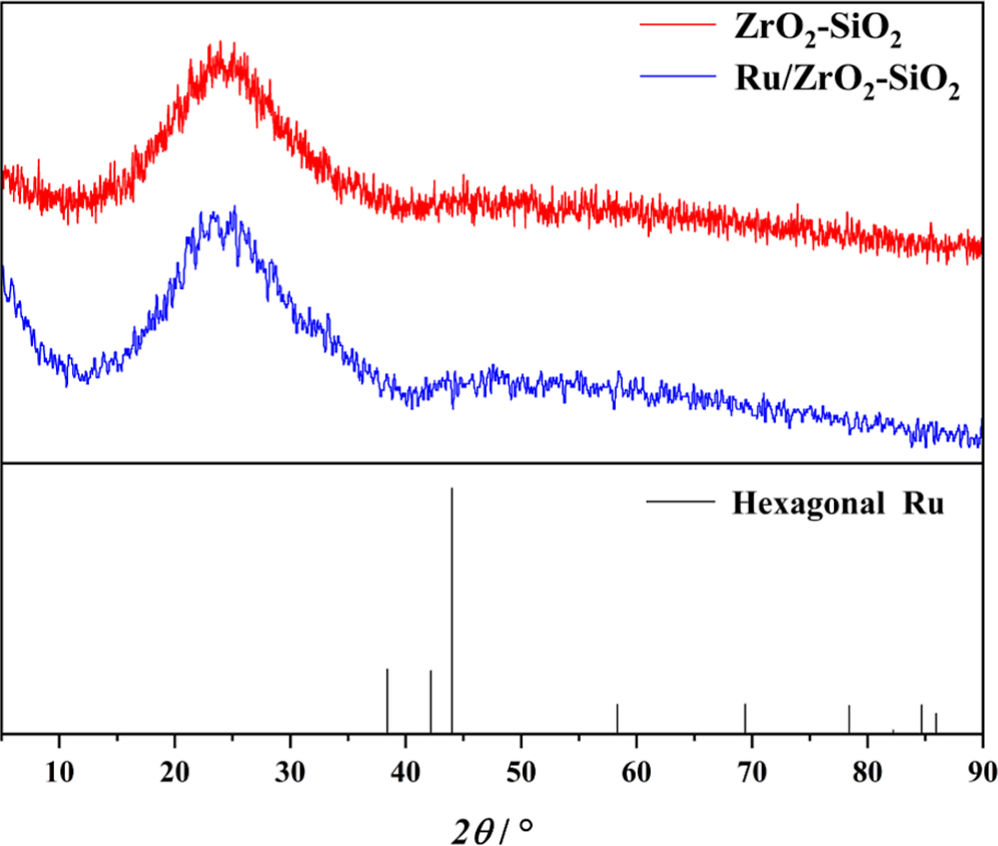
XRD patterns of Ru/ZrO_2_–SiO_2_ and ZrO_2_–SiO_2_.

The X-ray diffraction (XRD) analysis was conducted in order to identify the crystal structure of the catalysts. The composite oxide carrier ZrO_2_–SiO_2_ used in this study consisted of amorphous SiO_2_ and crystalline ZrO_2_ in a molar ratio of 10:1. Moreover, the loading amount of elemental ruthenium on ZrO_2_–SiO_2_ was only 5‰, making it difficult to detect in XRD analysis. Consequently, as depicted in Figure 2, the XRD pattern prominently featured the amorphous phase. The low loading rate resulted in less obvious Ru crystal peaks. Normally, metallic Ru(0) crystals exhibited a hexagonal shape, with the strongest peak (1,0,1) lattice plane at 2θ of 44°, and the second strongest peaks (1,0,0) and (0,0,2) lattice planes at 2θ of 38° and 42°, respectively.^33^ A detailed comparison of the XRD curves revealed a slight upward shift around 44° for Ru/ZrO_2_–SiO_2_ compared to ZrO_2_–SiO_2_, indicating the successful loading of Ru metal on the carrier. Nevertheless, on account of the low content of Ru, no obvious peak of crystal Ru metal was observed. The XRD curves showed slightly less fluctuation after loading, suggesting that the loading of Ru improved the crystallinity of the carrier, which was attributed to the forces between the surface-loaded Ru and the crystals, leading to a more stable internal structure of the ZrO_2_–SiO_2_ material.^34,35^

#### 3.1.3 Surface and Porous Structure of the Ru/ZrO_2_–SiO_2_ Catalyst

**Figure 3:**
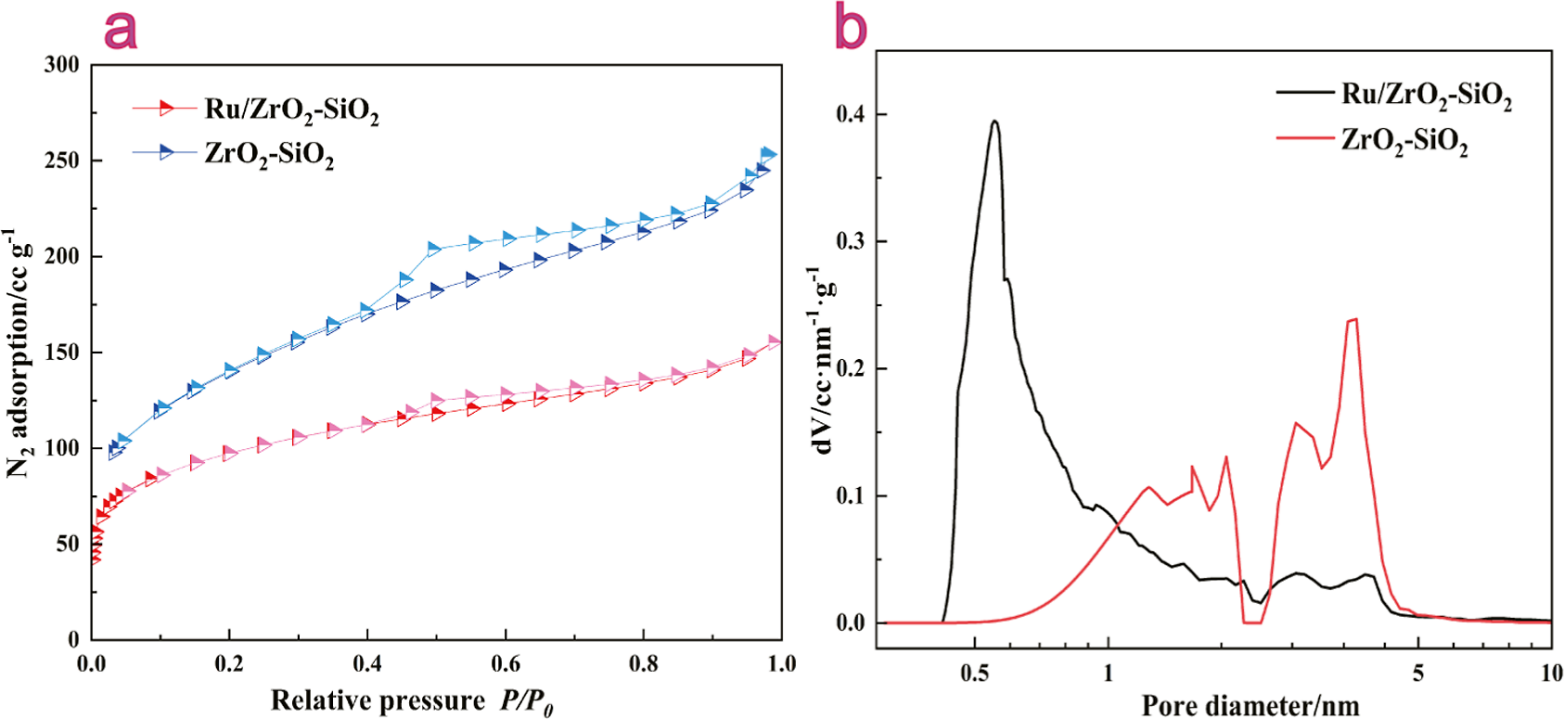
(a) Adsorption curves and (b) pore diameter distribution of Ru/ZrO_2_–SiO_2_ and ZrO_2_–SiO_2_.

The BET analysis was conducted to investigate the N_2_ adsorption–desorption curves and pore size distribution of the composite oxide carriers before and after Ru loading. The overall adsorption–desorption curve of Ru/ZrO_2_–SiO_2_, presented in Figure 3a, exhibited a mixed I-shaped and IV-shaped pattern with H4 hysteresis loops, indicating a combination of micro- and mesoporous structures, with narrow fracture pores dominating the solid structure.^36^ The pore structure of ZrO_2_–SiO_2_ was not significantly altered after Ru loading. Specifically, at lower relative pressures (*P*/*P*_0_ close to 0), the adsorption curve of Ru/ZrO_2_–SiO_2_ showed a steeper slope, indicating an increase in micropores and a conversion of some mesopores into micropores due to the partitioning of smaller Ru particles within the original carrier pores.^37^ Contrary to the increase in micropores, the mesopores exhibited a more pronounced change, characterized by a sharp weakening of the hysteresis loop and a decrease in mesopore content owing to the partitioning of pore size by the Ru particles, leading to the conversion of some mesopores into micropores.^38^ As the relative pressure approached 1, the loading amount gradually increased without an adsorption termination plateau, indicating a weakened multilayer adsorption after mesopore capillary coalescence for the introduction of Ru particles, resulting in a more compact stacking of Ru/ZrO_2_–SiO_2_ compared to ZrO_2_–SiO_2_, leading to the disappearance of some macropores in the composite oxides.

The analysis of the pore size distribution, as depicted in Figure 3b, confirmed a decrease in mesopores in Ru/ZrO_2_–SiO_2_ compared to ZrO_2_–SiO_2_. The micropores in Ru/ZrO_2_–SiO_2_ were predominantly distributed around 0.6 nm, which was smaller than those in ZrO_2_–SiO_2_ owing to the splitting of the original pores during Ru particle filling. Additionally, the specific surface area analysis revealed that Ru/ZrO_2_–SiO_2_ had micropores, mesopores, and a total specific surface area of 175.89 , 63.94 , and 333.39 m^2^ g^–1^, respectively, while ZrO_2_–SiO_2_ had micropores, mesopores, and a total specific surface area of 159.06, 143.06, and 416.86 m^2^ g^–1^, respectively. The observed changes in the total specific surface area could be ascribed to the disappearance of larger pores and the agglomeration of composite oxide carrier particles during the loading process. The BET analysis indicated modifications in the pore structure and surface area of Ru/ZrO_2_–SiO_2_ after Ru loading, potentially influencing its performance in biocrude production during the HTL process.

The specific surface areas of Ru/ZrO_2_–SiO_2_ at different Ru loading ratios are presented in Table 3. It can be observed that when Ru was loaded, the specific surface area of micropores increased, while the specific surface area of mesopores decreased. This can be ascribed to the partitioning effect of Ru particles during the loading process. Ru particles entered into the pores and filled some space of ZrO2–SiO2, reducing the area of mesopores and further resulted in the conversion of some mesopores into micropores. For a Ru loading ratio of 1‰, the specific surface area of micropores increased from 159.06 to 216.48 m^2^ g^–1^. With increasing Ru loading ratio, the specific surface area of micropores fluctuated between 170 and 190 m^2^ g^–1^ in the range of 2–5‰. The specific surface area of mesopores was 78.47 m^2^ g^–1^ for a Ru loading ratio of 1‰ and fluctuated between 60 and 73 m^2^ g^–1^ as the loading ratio increased.

**Table 3:** Specific Surface Areas of Ru/ZrO_2_–SiO_2_ at Different Ru Loading Ratios

surface area/m^2^ g^–1^	Ru loading ratio/‰					
	ZrO_2_–SiO_2_	1	2	3	4	5
micropores	159.06	216.48	186.96	178.28	188.56	175.89
mesopores	143.06	78.47	72.18	65.03	68.38	63.94
total	461.86	365.46	350.70	345.71	359.42	333.39

#### 3.1.4 Micro Morphology of the Ru/ZrO_2_–SiO_2_ Catalyst

**Figure 4:**
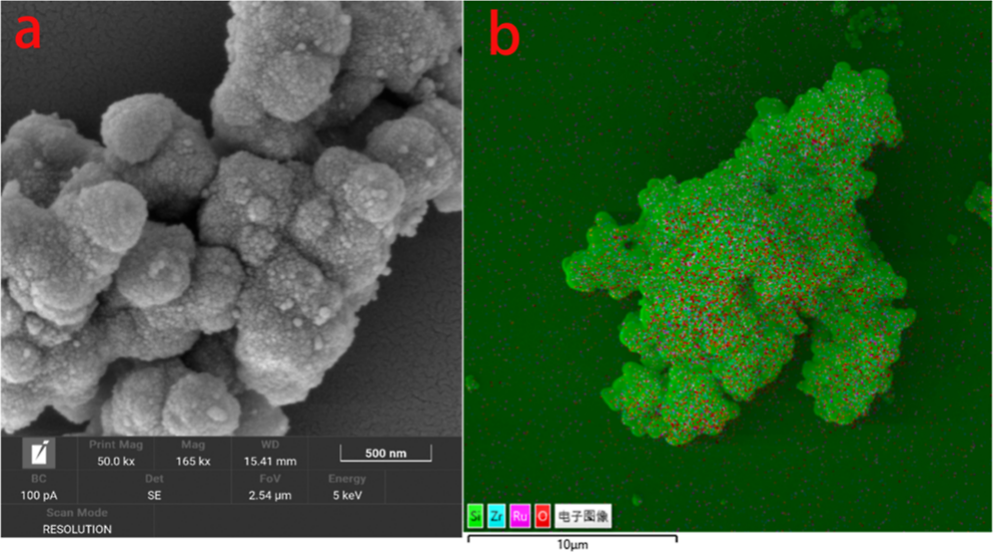
SEM (a) and mapping (b) results of Ru/ZrO_2_–SiO_2_.

As shown in Figure 4, the Ru/ZrO_2_–SiO_2_ particles were regular and spherical. The Ru loading ratio of 5‰ resulted in an even distribution of Ru color dots on the composite oxide carrier, proving that Ru dispersed evenly on the carrier.

### 3.2 Effect of Ru Loading Ratio on the Catalytic HTL of *Spirulina*

#### 3.2.1 Biocrude Yield

**Figure 5:**
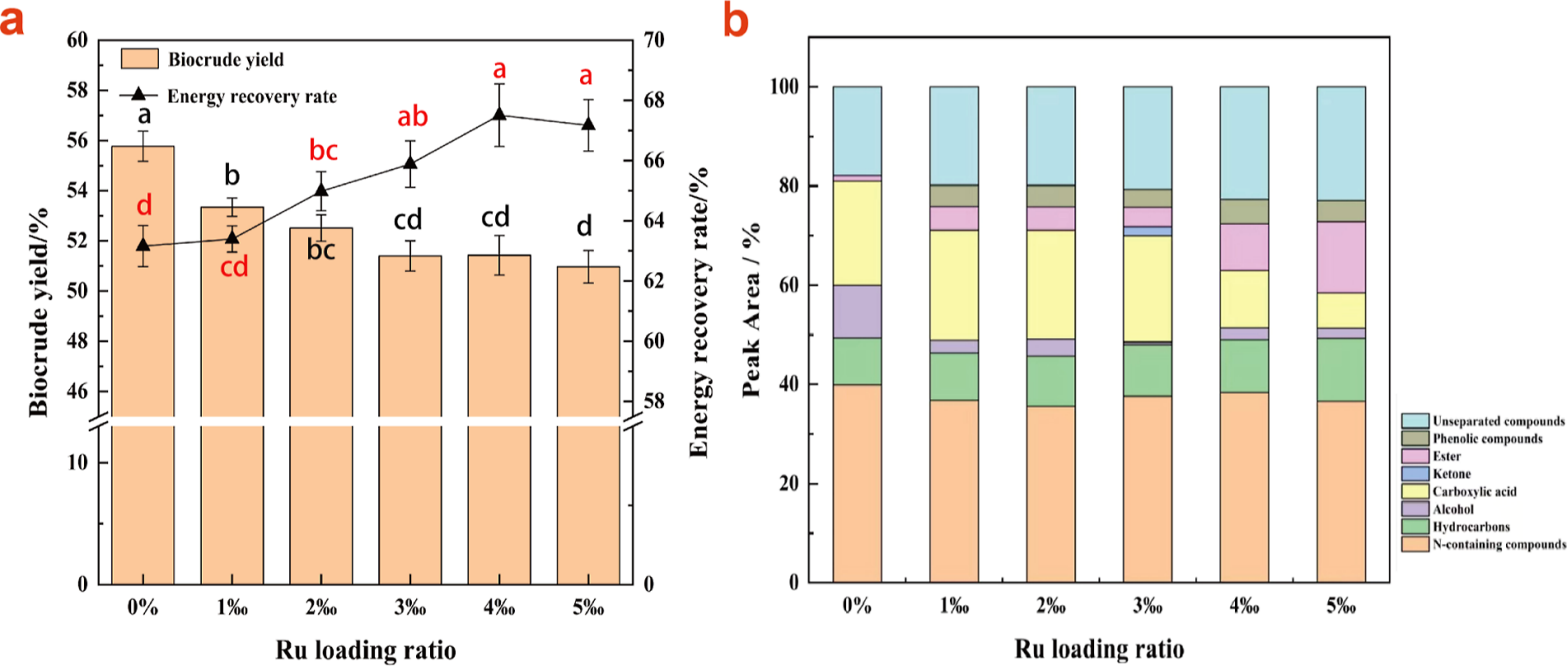
Effect of different Ru loading ratios on biocrude yield, ER rate (a) and biocrude composition (b) (the black letters on the column indicate significant differences in biocrude yield, while the red letters on the line symbols denote significant differences in ER rates).

To control the cost of the catalyst, the Ru loading ratio was kept relatively low despite its excellent catalytic performance. As shown in Figure 5a, the biocrude yields decreased gradually as the Ru loading ratio increased from 1‰ to 5‰, with values of 53.34%, 52.51%, 51.40%, 51.42%, and 50.97%, respectively. These values were significantly lower compared to the ZrO_2_–SiO_2_ carrier (55.77%, *P* < 0.05). The gap in biocrude yield between ZrO_2_–SiO_2_ and 1‰ loading ratio group was particularly pronounced, and then the decrease was gradual as the Ru loading ratio increased and reached a stable value at a loading ratio of 3‰ by way of the changes in the pore structure of the composite oxide carrier caused by the filling of Ru particles, which might have a negative impact on the HTL process. Comparing the changes in specific surface area, as discussed in Section 3.1.3, with the biocrude yield, a positive correlation can be observed between the specific surface area of mesopores and the biocrude yield to some extent. The biocrude yield stabilized when the specific surface area of mesopores reached a stable value.

#### 3.2.2 Element Analysis

The elemental analysis of biocrude at different Ru loading ratios is presented in Table 4. It was observed that the S content of the biocrude decreased significantly after Ru loading due to the catalytic effect of Ru, which promoted the hydrolysis of disulfide bonds in proteins, resulting in their redistribution into other phases and inhibiting sulfur into the biocrude.^39^ Reducing the sulfur content would be beneficial not only for improving the quality of the biocrude but also for facilitating the subsequent upgrading process, particularly when using Ru-based catalysts, which are susceptible to poisoning by sulfur.^40^ As the loading ratio of Ru increased, the H content gradually increased, while the O content decreased. The elemental composition reached a stable state when the loading ratio reached 4‰. This indicated that Ru/ZrO_2_–SiO_2_ had a catalytic effect on the hydrodeoxygenation of biocrude during the HTL process of *Spirulina*, which likely involved the hydrogenation reduction of carboxylic acids, aldehydes, and alcohols. The increase in C content along with the reduction of biocrude yield after hydrodeoxygenation suggested that more carbon was concentrated into the biocrude phase. Fluctuations in N content suggested that Ru/ZrO_2_–SiO_2_ did not have a significant catalytic effect on N-containing functional groups. The optimal elemental content and HHV of the biocrude were achieved at a Ru loading ratio of 5‰, with N, C, H, S, and O contents of 9.32%, 64.17%, 7.93%, 0.61%, and 17.97%, respectively, and an HHV of 30.63 MJ kg^–1^.

**Table 4:** Element Analysis of Biocrude at Different Ru Loading Ratios

Ru loadingratio/‰	element content/wt %					HHV/MJ kg^–1^
	N	C	H	S	O^a^	
ZrO_2_–SiO_2_	7.97	57.04	7.12	1.05	26.82	26.32
1	9.16	58.99	7.42	0.55	23.88	27.62
2	8.48	61.05	7.67	0.55	22.25	28.76
3	8.91	62.65	7.85	0.64	19.95	29.79
4	9.64	64.01	7.90	0.43	18.02	30.51
5	9.32	64.17	7.93	0.61	17.97	30.63

a Calculated by differences.

#### 3.2.3 Energy Recovery Rate

As the Ru loading ratio increased, the biocrude yield gradually decreased, while the HHV increased. However, determining the optimal loading ratio was challenging. Therefore, the ER rate was used as an evaluation parameter for the HTL process. As shown in Figure 5a, the ER rates for Ru loading ratios of 1 to 5‰ were 63.40%, 64.98%, 65.89%, 67.51%, and 67.17%, respectively, while it was 63.16% for the bare ZrO_2_–SiO_2_ carrier. Aside from the 1‰ loading ratio, all other treatment groups exhibited significant improvement in ER rates compared to ZrO_2_–SiO_2_ (*P* < 0.05). This indicates that Ru loading could improve the ER rate. The limited amount of Ru in the 1‰ treatment group may explain the lack of significant differences compared to the bare carrier. Furthermore, as the loading ratio of Ru increased, the ER rate initially increased and then gradually stabilized. The ER rate of the HTL of *Spirulina* reached stability at a loading ratio of 4‰, suggesting that this ratio could be considered optimal.

#### 3.2.4 GC–MS Analysis

The gas chromatography–mass spectrometry(GC–MS) method was utilized to analyze the molecular composition of biocrude samples, and the results of the biocrude at different Ru loading ratios are presented in Figure 5b. We adopted a prioritization method for categorization of the biocrude components, based on the predominant functional group present in each component. It could be observed that the content of unseparated compounds in the biocrude increased with Ru loading, indicating an increase in the amount of more complex compounds. This suggested that the depolymerization of biomass macromolecules was hindered during the HTL process. Furthermore, as the Ru loading ratio increased, the content of complex compounds increased, while the biocrude yield decreased, indicating that the Ru particles weakened the depolymerization of macromolecules. The appearance of phenolic compounds after Ru loading was a result of the decomposition of lignin in *Spirulina* caused by Ru particles, resulting in the incorporation of the products into the biocrude phase. The N-containing compounds exhibited irregular changes with the loading of Ru particles, which could be influenced by variations in both the biocrude yield and the absolute amount of N-containing compounds.

Figure 5b also illustrates the carboxylic acid content of the 1‰ Ru loading group, which was higher than that of the control group with bare ZrO_2_–SiO_2_. As the Ru loading ratio increased, the carboxylic acid content gradually decreased, while the ester content increased. However, the combined content of carboxylic acid and ester still decreased due to the hydrodeoxygenation effect of Ru/ZrO_2_–SiO_2_ on carboxylic acid substances. A small amount of ketones appeared at a loading ratio of 3‰, which was partially produced through the reaction of carboxylic acids or esters. The alcohols were significantly reduced compared to the control group, probably because Ru/ZrO_2_–SiO_2_ promoted the esterification of carboxylic acids and alcohols in the biocrude. Additionally, by dint of the hydrodeoxygenation of carboxylic acids and alcohols, the content of hydrocarbons increased with the increasing Ru loading ratio. Overall, the content of hydrocarbons increased while carboxylic acids and esters decreased. The content of N-containing compounds did not exhibit significant changes, consistent with the results from the elemental analysis.

### 3.3 Effect of Ru/ZrO_2_–SiO_2_ Ratio on Catalytic HTL of *Spirulina*

#### 3.3.1 Biocrude Yield

**Figure 6:**
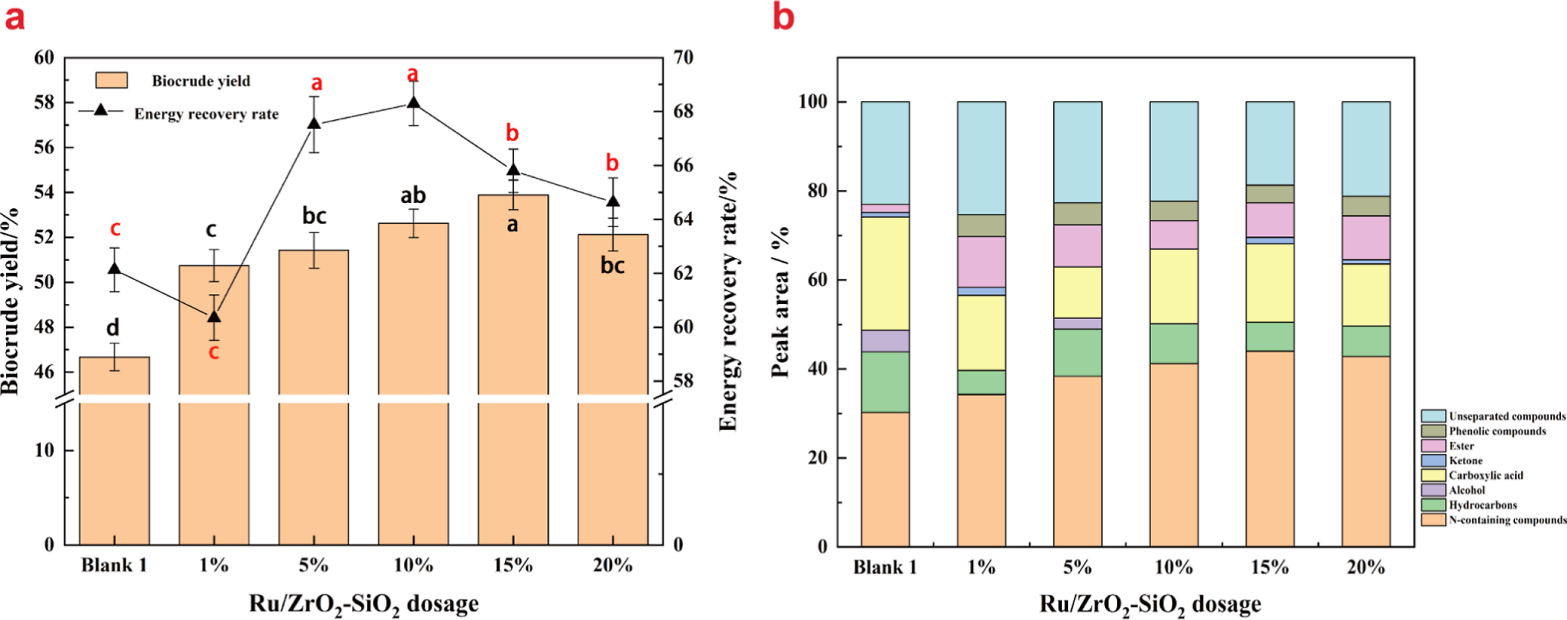
Effect of Ru/ZrO_2_–SiO_2_ dosage on biocrude yield, ER rate (a) and biocrude composition (b) (the black letters on the column indicate significant differences in biocrude yield, while the red letters on the line symbols denote significant differences in ER rates).

The effect of different amounts of Ru/ZrO_2_–SiO_2_ on the yield of biocrude produced from *Spirulina* is depicted in Figure 6a, with the blank 1 group serving as the control without a catalyst. Compared to the blank 1 group, the addition of various amounts of Ru/ZrO_2_–SiO_2_ resulted in significant improvement in biocrude yields owing to the favorable pore structure and acid–base properties of Ru/ZrO_2_–SiO_2_ (*P* < 0.05), which facilitate the conversion of algal biomass macromolecules into biocrude. The biocrude yields were 50.74%, 51.42%, 52.62%, 53.89%, and 52.12% for catalyst ratios of 1%, 5%, 10%, 15%, and 20%, respectively. When the catalyst ratio was below 15%, the biocrude yield increased with increasing catalyst ratios owing to the presence of more micropores and mesopores provided by the catalyst, which catalyzed various reactions during the HTL process.^41^ When the catalyst ratio exceeded 15%, however, the biocrude yield decreased, as a result of the catalyst overload and competition between the pores for the adsorption of biomass molecules, leading to the hindering of catalytic reactions.

#### 3.3.2 Element Analysis

The element analysis of biocrude produced at various Ru/ZrO_2_–SiO_2_ ratiosis presented in Table 5. Compared to the blank 1 group, the addition of the catalyst resulted in a slight decrease in the C and H contents and HHV of the biocrude, while the O content increased. This was because the biocrude yield increased significantly after catalyst addition, resulting in the incorporation of oxygenated polar substances from the aqueous phase into the biocrude phase through a series of reactions. Without considering the hydro-deoxygenation effect of the catalyst, the increase in biocrude yield would inevitably lead to a decline in its quality. The element analysis results at different catalyst ratios showed irregular fluctuations in the N, C, H, S, and O contents because the catalyst carrier primarily contributed to the increase in biocrude yield, while the active component, Ru, promoted hydrodeoxygenation, resulting in fluctuations in the content of each element. The lowest N content (7.95%) was observed at a 15% ratio, the highest C content (64.35%) at 10%, the highest H content (7.90%) at 5%, the lowest O content (18.02%) at 5%, and the highest HHV (30.51 MJ kg^–1^) at 5%. However, on account of the significant variation in yield, it was challenging to determine the optimum dosage ratio based on HHV.

**Table 5:** Element Analysis of Biocrude at Different Ru/ZrO_2_–SiO_2_ Dosages

Ru/ZrO_2_–SiO_2_ dosage/%	element content/wt %					HHV/MJ kg^–1^
	N	C	H	S	O^a^	
blank 1	8.93	64.41	8.10	0.74	17.82	30.94
1	8.97	59.28	7.31	0.76	23.68	27.64
5	9.64	64.01	7.90	0.43	18.02	30.51
10	8.16	64.35	7.67	0.57	19.25	30.16
15	7.95	61.32	7.28	0.92	22.53	28.38
20	8.52	62.78	7.17	0.41	21.12	28.82

a Calculated by differences.

#### 3.3.3 Energy Recovery Rate

The ER rate of biocrude at different Ru/ZrO_2_–SiO_2_ ratios is presented in Figure 6a. The ER rate of biocrude increased with increasing amounts of catalyst when the ratio was below 10%, but showed a decreasing trend when the ratio exceeded 10%. Hence, the 10% treatment group achieved the highest ER rate of 68.92%. Compared to the blank 1 group, the ER rate was significantly lower at a 1% dosage rate, primarily due to the substantial improvement in biocrude yield by the catalyst carrier, while Ru had only a slight effect on biocrude quality. With the exception of the 1% treatment group, the ER rates of the catalyst addition groups were significantly higher than that of the blank group (*P* < 0.05), demonstrating the effectiveness of Ru/ZrO_2_–SiO_2_ in enhancing ER rates during the HTL of *Spirulina*. The increase in ER rate at 10% compared to the 5% treatment group could be put down to changes in biocrude yield, despite a slight decrease in HHV. The decrease in ER rate in the 20% treatment group was with a simultaneous decrease in biocrude yield and HHV, indicating inhibition of the hydrodeoxygenation effect of the active component, possibly resulting from excess active component competing for reactions.

#### 3.3.4 GC–MS Analysis

The GC–MS analysis of biocrude at different Ru/ZrO_2_–SiO_2_ ratios is shown in Figure 6b. The results revealed that at a low catalyst dosage (1%), owing to the insufficient catalytic activity of the low dosage in depolymerizing macromolecules, there were higher amounts of unseparated compounds in the biocrude, resulting in a higher content of complex compounds in the biocrude. With an increase in catalyst dosage, the content of unseparated compounds initially decreased and then increased due to the dual effects of the catalyst, promoting both the hydrolysis of large molecules and the condensation of small molecules. At low dosages, the catalyst primarily promoted the condensation of small molecules, while at high dosages, it mainly facilitated the hydrolysis of large molecules. The content of unseparated compounds exhibited a negative correlation with the biocrude yield, consistent with the result of 3.2.4. The addition of the catalyst led to an increase in N-containing compounds compared to the blank 1 group, indicating the catalyst’s ability to catalyze the polymerization of small amino acids into the oil phase. The content of N-containing compounds gradually increased and then stabilized with increasing Ru/ZrO_2_–SiO_2_ dosage by way of the competition between catalysts at high dosages, limiting the catalytic activity. Furthermore, a positive correlation was observed between the content of N-containing compounds and the biocrude yield. This was consistent with our previous study,^14^ in which we found that suitable catalyst could promote the migration of N element from aqueous phase to oil phase, which accounted for the improvement of biocrude yield.

The total amount of carbonyl compounds (carboxylic acids, esters, and ketones) in the biocrude decreased to some extent with the addition of Ru/ZrO_2_–SiO_2_, suggesting the hydrodeoxygenation effect of Ru/ZrO_2_–SiO_2_ on carbonyl compounds. The content of carbonyl compounds fluctuated with increasing Ru/ZrO_2_–SiO_2_ dosage because of the simultaneous catalytic hydrogenation effect and the improvement in biocrude yield by the catalyst. The GC–MS and element analysis demonstrated a positive correlation between the content of carbonyl compounds and the oxygen element, indicating that changes in carbonyl compounds were the primary cause of O fluctuation and contributed to the variation in HHV. In addition , the content of hydrocarbons was negatively correlated with carbonyl compounds, suggesting that part of hydrocarbons could be produced by decarboxylation of organic acids, which have been proven by others.^42^

### 3.4 Implications and Outlook

HTL is a promising method for converting biomass to bioenergy. However, the conversion performance is unsatisfactory under normal conditions. So catalysts are widely employed in the HTL process to improve biocrude yield and quality. In our previous study, we conducted HTL of *Spirulina* using ZrO_2_–SiO_2_ and achieved higher biocrude yield while reducing the biocrude quality with the characteristic of higher O content and lower C and H content. Herein, the Ru/ZrO_2_–SiO_2_ catalyst was prepared and applied in the HTL of *Spirulina* to improve biocrude quality. As mentioned above, the loading of Ru on composite oxide actually improved C and H content while decreasing the O content, resulting in higher HHV and ER rate. This was consistent with another research, in which the authors investigated the effect of the Ru/C catalyst on HTL of microalgae. They found that the Ru/C catalyst could improve C and H content significantly and decrease the content of the O atom at the same time, with the HHV increase from 28.08 to 35.60 MJ kg^–1^. This may be caused by a series of reactions, such as catalytic hydrodeoxygenation, decarboxylation, decarbonylation, and dehydration.^43^ In addition, Ru-based catalysts are found effective in denitrification and desulfuration in HTL. For example, Xu et al. studied biocrude upgrading with various catalysts; the results showed that the Ru/C catalyst could reduce the content of N and S simultaneously, the N content decreased from 7.7% to 2.6%, while S decreased from 0.76% to undetectable.^44^

Apart from Ru, other metals, such as Ni, Pt, Pd, and so on, were also used in catalytic hydrothermal liquefaction. As to the carrier, active carbon, metallic oxide, and molecular sieves are commonly used. So the combination of various metals with carriers and their application in HTL can be a promising method in the future.

## 4 Conclusions

This study explored the preparation of the Ru/ZrO_2_–SiO_2_ catalyst and its application in the HTL of *Spirulina* for biocrude production. Characterization of Ru/ZrO_2_–SiO_2_ was conducted, and the impact of different loading ratios of Ru particles and Ru/ZrO_2_–SiO_2_ dosage on biocrude production were investigated. The findings are as follows:

1. Ru particles were successfully deposited on the carrier and improved the crystallinity and internal structure of ZrO_2_–SiO_2_.
2. The biocrude yield gradually decreased as the loading ratio of Ru particles increased, stabilizing at a loading ratio of 3‰. The highest ER rate of 67.51% was achieved at a loading ratio of 4‰. GC–MS results demonstrated the hydro-deoxygenation effect of Ru.
3. The biocrude yield initially increased and then decreased with increasing Ru/ZrO_2_–SiO_2_ addition, peaking at 53.89% with a dosage of 15%. Similarly, the ER rate exhibited the same trend, reaching a maximum of 68.29% at a catalyst dosage of 10%.

In conclusion, the loading of Ru particles onto the composite oxide carrier enhanced the stability and catalytic performance of the catalyst. The findings of this study provide valuable insights into the development of efficient catalysts and process optimization in the HTL of *Spirulina* for biocrude production.
